# Reliability and validity of the Japanese version of the Pediatric Quality of Life Inventory Infant Scales

**DOI:** 10.1186/s41687-022-00416-3

**Published:** 2022-01-29

**Authors:** Iori Sato, Takafumi Soejima, Mari Ikeda, Kyoko Kobayashi, Ami Setoyama, Kiyoko Kamibeppu

**Affiliations:** 1 Division of Health Sciences and Nursing, Department of Family Nursing, Graduate School of Medicine, The University of Tokyo, Hongo, Bunkyo-ku, Tokyo, Japan; 2 Division of Family Health Care Nursing, Graduate School of Health Sciences, Kobe University, Tomogaoka, Suma-ku, Kobe, Japan; 3 Department of Child Health Nursing, Graduate School of Nursing Science, St. Luke’s International University, Akashi-cho, Chuo-ku, Tokyo, Japan; 4 QOL Research Center for Children and Family, Tokyo, Japan

**Keywords:** Infant, Japan, Parents, PedsQL™, Quality of life, Questionnaire

## Abstract

**Background:**

PedsQL Infant Scales (PedsQL-I) are used to assess parent-reported health-related quality of life for children younger than 2 years. We determined the feasibility, reliability, and validity of the Japanese version of the PedsQL-I.

**Methods:**

A total of 183 participants (parents) with infants aged 1–30 months were recruited from 8 day care centers and one pediatric clinic. Participants completed the PedsQL-I (infants aged 1–18 months), the PedsQL-I and the PedsQL-Toddler version (infants aged 19–30 months), and the Kessler-6 psychological distress scale (all participants). We determined feasibility, internal consistency, test–retest reliability, concurrent validity, convergent and discriminant validity, known-groups validity with regard to acute and chronic illness, and relative and transitional validity with PedsQL-Toddler for the use in infants aged 25–30 months.

**Results:**

All subscales were internally consistent (Cronbach’s alpha for 1–12 months: 0.88–0.98 and for 13–24 months: 0.85–0.97); test–retest reliability was acceptable (intra-class correlation coefficients > 0.40); and all scales were concurrently valid with the PedsQL-Toddler version (Pearson’s product-moment correlation coefficient for the total score = 0.74). The scales’ convergent and discriminant validity were acceptable (scaling success rate > 80%). Validation for known-groups showed that the Physical Health Summary score was sensitive to acute and chronic disease, the Psychosocial Health Summary score was sensitive to neither acute nor chronic disease, and the total score was sensitive to acute disease. Relative validity showed a ratio of 1.74 for the squared t values for the total score.

**Conclusions:**

The PedsQL-I is suitable for assessing health-related quality of life in infants aged 1–24 months in prospective studies.

**Supplementary Information:**

The online version contains supplementary material available at 10.1186/s41687-022-00416-3.

## Background

In recent years, it has been accepted by medical practitioners and researchers that patient-reported outcomes (PROs) should be implemented into clinical practice and research, in addition to objective clinical outcomes such as laboratory values. Health-related quality of life (HRQOL), which is a PRO, assesses some aspects of quality of life affected by diseases and their treatment [1–3]. Aspects such as the level of physical, psychological, and social well-being are also called generic HRQOL. The generic HRQOL can be compared between individuals with and without health problems. Hence, it can be used for the health evaluation of various populations [4, 5]. Therefore, HRQOL has established itself as an important outcome in clinical research, clinical practice, and community health [6–8].

Self-report is the standard method of measuring HRQOL. The same is true for children, but HRQOL for children who are too young to self-report or cognitively impaired requires parent-report [9]. There are several scales that provide parent-report for HRQOL for children older than 2 years (e.g., Pediatric Quality of Life Inventory (PedsQL) Generic Core Scales [10]), but HRQOL scales available for infants younger than 2 years are limited. In addition to the PedsQL Infant Scales (PedsQL-I) [11], the TNO/AZL Preschool Children Quality of Life Questionnaire (TAPQOL) [10, 12] and the Infant and Toddler Quality of Life Questionnaire (ITQOL) [13, 14] are used. However, the TAPQOL is not available until after the first year of life, and the ITQOL has 103 items, making it burdensome for the respondent. The PedsQL-I can be used from the first month of life, and having only a small number of items (36 items (1–12 months) or 45 items (13–24 months)), it is considered to be a simple and highly useful HRQOL scale.

One of the advantages of the PedsQL-I is its suitability for longitudinal HRQOL tracking: the two-factor structure of the PedsQL-I with physical and psychosocial health summary scores is similar in structure to the PedsQL Generic Core Scales [5, 10], which can be used from age 2–25 [15, 16]. Therefore, it can be used with conceptual consistency in longitudinal quality of life studies that follow patients for several years. The purpose of the present study was to develop a Japanese version of the PedsQL-I and to investigate its feasibility, reliability, and validity. In particular, we examined the relative and transitional validity of the PedsQL-I at the age boundary to determine its usefulness in longitudinal follow-up studies that continue beyond age two.

## Methods

### Scale development

Dr. James W. Varni (JWV) permitted the translation of the PedsQL-I [11] into Japanese. Two researchers separately translated the original scale using an approved translation procedure [17]. We reconciled these translations into a single Japanese version, preserving the wording between this scale and the Japanese version of PedsQL Generic Core Scales [18]. This preservation is needed because consistency between PedsQL-I and the PedsQL Generic Core Scales Toddler version (PedsQL-T) is necessary for tracking HRQOL over time. A native English translator, who was blinded to the original version, back-translated the reconciled version into English. Seven Japanese health professionals (pediatric nurses, clinical psychologists, and a nurse-midwife, including bilingual/bicultural researchers) compared the back-translated version with the original English version and made minor amendments to the reconciled Japanese version to produce a pilot questionnaire [19].

Ten native Japanese-speaking parents who nurtured infants aged 1–24 months participated in the pilot test between November 2013 and January 2014 [19]. The data obtained from the pilot test were used to produce a final Japanese version of the PedsQL-I. No words or phrases required modification after the pilot test. In addition, JWV confirmed the conceptual and linguistic equivalence between the Japanese version and the original version.

### Study population

Parents with children aged 1–30 months were recruited from eight day care centers and one pediatric clinic located in Tokyo using distributed flyers. Recruitment was carried out between July and September 2014. Parents whose ability to read, understand and communicate in Japanese was judged as insufficient by the day care center/pediatric clinic staff were excluded. The present study included parents with children aged 25–30 months to examine transitional validity at the age boundary.

We calculated sample size based on known-group validity. Assuming a 1:5 ratio between those with and without a disease, it was calculated that 38 infants and 190 infants, respectively, were needed to detect a moderate difference (effect size d = 0.5) with a power of 0.8 at a significance level of 0.05.

### Procedure

The study collaborators distributed the questionnaires and return envelopes. Participants received a detailed information sheet informing them of the content and extent of the study, and their completing the questionnaire was taken as providing informed consent. The questionnaires were either returned by mail or collected from designated questionnaire return boxes placed in the day care centers and the pediatric clinic. Parents with children aged 1–24 months were informed of the retest procedure during the first test. Participants who consented to the retest were sent retest questionnaires in self-addressed envelopes 1–2 weeks after the initial questionnaire, asking participants to return the retest within a week of receipt. Upon completion of the study, a summary of the study results was sent to the heads of the day care centers and the pediatric clinic.

### Measurement

The PedsQL-I has two age-appropriate versions, for ages 1–12 months and 13–24 months [11]. The 1–12 months version and the 13–24 months version include the same five scales (36 and 45 items, respectively): Physical Functioning (6 and 9 items, respectively), Physical Symptoms (both include 10 items), Emotional Functioning (both include 12 items), Social Functioning (4 and 5 items, respectively), and Cognitive Functioning (4 and 9 items, respectively). The PedsQL-I also includes the Physical Health Summary score (the mean of the item scores included in the Physical Functioning and Physical Symptoms Scales) and the Psychosocial Health Summary score (the mean of the item scores included in the Emotional, Social, and Cognitive Functioning Scales). Respondents are asked to describe the extent to which each item had troubled their children over the past 1 month (the PedsQL’s standard recall period). A 5-point Likert response scale is used (0 = never a problem; 1 = almost never a problem; 2 = sometimes a problem; 3 = often a problem; 4 = almost always a problem). Items are reverse-scored and linearly transformed to a 0–100 scale (0 = 100, 1 = 75, 2 = 50, 3 = 25, 4 = 0), where higher scores indicate a better HRQOL. Scale scores and the total score are computed as the sum of the items divided by the number of items answered. If more than 50% of the items are missing or incomplete, the scale score is not computed.

PedsQL-T consists of 21 items that belong to one of the four following scales: Physical Functioning (8 items), Emotional Functioning (5 items), Social Functioning (5 items), and School Functioning (3 items) [18]. The PedsQL-T also includes the Physical Health Summary score (same as the Physical Functioning scale) and the Psychosocial Health Summary score (the mean of the item scores included in the Emotional, Social, and School Functioning Scales). These scale scores and the total score are computed as the sum of all the items on the PedsQL-I in a similar manner.

Kessler-6 (K6) consists of six items and was used to screen parents for psychological distress [20]. Respondents rate how often they felt (1) nervous, (2) hopeless, (3) restless or fidgety, (4) so depressed that nothing could cheer them up, (5) that everything was an effort, and (6) worthless over the past one month. A 5-point Likert response scale was used (0 = none of the time; 1 = a little of the time; 2 = some of the time; 3 = most of the time; 4 = all of the time). Responses to the six items were summed up to yield a K6 score between 0 and 24, with higher scores indicating a greater tendency towards psychological distress.

Participants were asked about their age, gender, familial relations to their child, and economic status. They also answered the items regarding characteristics of their children (age, gender, place where a child spends daytime, and presence of acute, chronic illness and/or other disease). We also added one question to the retest questionnaire: ‘Has a significant event affecting you happened since responding to the initial questionnaire?’.

Participants with children aged 1–18 months completed the PedsQL-I and participants with children aged 19–30 months completed the PedsQL-I and PedsQL-T. All participants answered the items of K6.

### Statistical analyses

All analyses were performed using IBM SPSS software, version 21 (SPSS, Inc., Chicago, IL, USA) and R version 3.2.1 (R Foundation for Statistical Computing, Vienna, Austria) [21]. The level of significance was set at 0.05.

Score distributions for the PedsQL-I were summarized as mean, standard deviation, minimum and maximum scores, and percentages of floor (0) and ceiling (100) scores, in children aged each 1–12 months and 13–24 months. We defined a high floor/ceiling effect as more than 20% and an especially-high floor/ceiling effect as more than 50%. We assessed correlations between subscales on PedsQL-I in children aged each 1–12 months and 13–24 months by calculating Pearson’s product–moment correlation coefficient.

Feasibility was determined based on the percentage of missing values. Independence of easily missed items was assessed by Cochran’s Q test.

Reliability was assessed based on internal consistency and test–retest reliability among children aged 1–24 months. Good internal consistency was defined as a Cronbach’s alpha exceeding 0.70. To determine test–retest reliability, intraclass correlation coefficients (ICC) between the initial test and retest scores in a one-way random effects model were calculated; an ICC value of 0.40 represented moderate, 0.60 good, and 0.80 high agreement [22]. A paired t test between the initial test and retest scores was used to check whether or not the PedsQL scores had changed.

Validity was assessed based on factorial validity, known-groups validity, concurrent validity, convergent and discriminant validity, and relative validity. Factorial validity and known-groups validity were assessed among children aged 1–24 months. Concurrent validity, and convergent and discriminant validity were assessed among children aged 18–24 months. Relative validity was assessed among children aged 18–30 months.

We used multi-trait analysis to assess factorial validity [23]. The Pearson’s product–moment correlation coefficients of an item of PedsQL-I with its own scale (corrected for overlap) and other scales were calculated. We used pairwise correlations because only participants with infants aged 13–24 months answered 3 items of the Physical Functioning Scale, one item of the Social Functioning Scale, and 5 items of the Cognitive Functioning Scale. We hypothesized that an item correlates more strongly with its own scale than other scales. Scaling success for any scale was also assessed as the number of convergent correlation coefficients significantly higher than the discriminant correlation coefficient divided by the total number of correlations.

To assess known-groups validity, we calculated the regression coefficients for the presence of acute or chronic illness with the Physical Health Summary, the Psychosocial Health Summary, and the total scores of the PedsQL-I using a linear mixed model. We controlled the child’s age, gender, the parent’s age, gender, and K6 score as fixed effects and the day care centers/pediatric clinic as a random effect in this model. The presence of acute illness was defined as a child’s having only acute illness, and the presence of chronic illness was defined as a child’s having chronic illness regardless of having acute illness. We hypothesized that PedsQL-I would demonstrate a lower Physical Health Summary score and total score under acute illness and a lower Physical and Psychosocial Health Summary score and total score under chronic illness. Before conducting the analysis, we checked the interaction between infant age and disease presence for both the 1–12 months and the 13–24 months version using a regression model, but the difference was not statistically significant. Therefore, we conducted this analysis using the combined versions.

To assess concurrent validity, we calculated Pearson’s product–moment correlation coefficients to confirm that the Physical Health Summary, the Psychosocial Health Summary, and the total score on the PedsQL-I were positively correlated with the same scale scores on the PedsQL-T. Correlation coefficients of 0.10 represent small, 0.30 medium, and 0.50 large correlations [24].

Convergent and discriminant validity were examined by calculating Pearson’s product–moment correlation coefficient between scales from PedsQL-I and PedsQL-T. We hypothesized that the correlation of the Physical Functioning and Physical Symptoms Scale of the PedsQL-I would be highest with the Physical Functioning Scale of PedsQL-T, the Emotional Functioning Scale of PedsQL-I with that of PedsQL-T, and the Social Functioning Scale of PedsQL-I with that of PedsQL-T.

Relative validity was assessed via the ratio of squared t values. PedsQL-I must be better able to show HRQOL differences between known-groups than PedsQL-T among infants. If PedsQL-T can differentiate HRQOL between known groups better than PedsQL-I, the suitability of PedsQL-I for infants would be questionable (because PedsQL-T can take the place of PedsQL-I). We calculated the t value of the difference of the PedsQL-I Scale scores between 19–24-month-old infants with and without any (acute, chronic, and/or other) diseases, and compared it with those of the difference of the PedsQL-T scores. A larger than 1 ratio of squared t values means that the PedsQL-I Scale is relatively-valid as HRQOL scale for infants compared to the PedsQL-T. As control experiments, we checked the ratio of squared t values among 25- to 30-month-old toddlers.

We wanted to answer the question of whether the results measured by the PedsQL-I were ready to be taken over by the PedsQL-T when an infant visiting a follow-up clinic (or participating in a longitudinal study) matured. The transitional validity was determined by estimating two linear models of the PedsQL score depending on age: the PedsQL-I Scale scores among 19–24-month-old infants and the PedsQL-T Scale scores among 25–30-month-old toddlers. From these models, we obtained two estimates of PedsQL score at 24.5 months old. We hypothesized that the two estimates coincided with each other and tested it by bootstrapping. Similarly, we also checked the score difference between 1–12-month-old and 13–24-month-old infants at 12.5 months old.

## Results

### Participant characteristics

We distributed questionnaires to 340 participants of which 190 (55.9%) were returned. Seven questionnaires were excluded because participants with children aged 1–12 months had mistakenly completed the PedsQL-I 13–24 months version. A total of 183 questionnaires were analyzed (53.8%). This sample consisted of 50 participants with infants aged 1–12 months, 95 participants with infants aged 13–24 months, and 38 participants with infants aged 25–30 months (Table 1). Thirty-four percent of children aged 1–12 months and 78.9% of children aged 13–24 months spent daytime at a day care center. Far more children had an acute (75/145, 52%, including cold, herpangina or otitis media) and/or chronic disease (18/145, 12%, including allergy, atopic dermatitis, or asthma) than we assumed.

**Table 1:** Socio-demographic characteristics

	*N* = 183								
	Infants aged 1–12 months (*n* = 50)			Infants aged 13–24 months (*n* = 95)			Infants aged 25–30 months (*n* = 38)		
	*n* or mean	% or SD	Range	*n* or mean	% or SD	Range	*n* or mean	% or SD	Range
*Child*									
Gender									
Male	27	54.0		47	49.5		23	60.5	
Female	23	46.0		48	50.5		15	39.5	
Place at where a child spends daytime									
Home	32	64.0		18	18.9		4	10.5	
Day care center	17	34.0		75	78.9		34	89.5	
Other	1	2.0		1	1.1		0	0.0	
Missing	0	0.0		1	1.1		0	0.0	
Presence of disease^a^									
Acute disease	25	50.0		50	52.6		27	71.1	
Chronic disease	4	8.0		14	14.7		6	15.8	
Other disease	4	8.0		13	13.7		3	8.9	
*Parents*									
Age (years)	35.7	5.3	23–52	35.8	5.4	22–55	35.3	4.3	26–45
Familial relations to children									
Mother	46	92.0		83	87.4		37	97.4	
Father	4	8.0		12	12.6		1	2.6	
Subjective opinion of current economic status									
Very affluent	6	12.0		3	3.2		0	0.0	
Reasonably affluent	15	30.0		23	24.2		8	21.1	
Normal	24	48.0		58	61.1		29	76.3	
Not reasonably affluent	4	8.0		9	9.5		1	2.6	
Not very affluent	1	2.0		2	2.1		0	0.0	
K6	3.0	3.7	0–24	3.2	3.6	0–15	2.2	3.4	0–14

K6, Kessler-6 questionnaire (range 0–24); SD, standard deviation

^a^Multiple answers allowed

### Scale descriptions

More than half of the participants reported the maximum possible score on the Social (82.0% and 72%) and Cognitive Functioning Scale (80.0% and 63%) for the 1–12 months and the 13–24 months version of the PedsQL-I Scales, respectively (Table 2). There was no floor effect in any scale. All Pearson’s correlation coefficients between subscales on PedsQL-I were significant in children aged both 1–12 months and 13–24 months (Table 3).

**Table 2:** Score distribution of the Japanese version of the PedsQL Infant Scales

	*N* = 145							
	*n*	Mean	SD	Min	Max	% Floor	% Ceiling	Cronbach’s α
*Infants aged 1–12 months*								
Physical functioning	49	89.2	20.9	25	100	0.0	58.0	0.93
Physical symptoms	49	88.6	17.3	25	100	0.0	22.0	0.88
Emotional functioning	49	86.8	16.4	27.1	100	0.0	20.0	0.93
Social functioning	50	94.8	17.6	0	100	2.0	82.0	0.98
Cognitive functioning	48	95.8	11.6	50	100	0.0	80.0	0.92
Physical health summary	49	90.6	12.6	40	100	0.0	18.0	0.93
Psychosocial health summary	49	89.9	13.9	37.5	100	0.0	2.0	0.94
Total	49	88.8	18.1	29.7	100	0.0	2.0	0.95
*Infants aged 13–24 months*								
Physical functioning	93	90.3	17.1	16.7	100	0.0	43.2	0.92
Physical symptoms	92	89.2	18.7	15	100	0.0	39.5	0.93
Emotional functioning	92	87.2	15.3	30	100	0.0	16.0	0.91
Social functioning	91	95.2	11.6	45	100	0.0	71.6	0.91
Cognitive functioning	90	95.2	9.1	63.9	100	0.0	63.0	0.85
Physical health summary	93	89.7	17.2	17.1	100	0.0	27.2	0.96
Psychosocial health summary	92	91.2	11.2	53.3	100	0.0	14.8	0.94
Total	92	90.6	12.9	41.7	100	0.0	11.1	0.97

SD, standard deviation

**Table 3:** Correlations between subscales on the Japanese version of the PedsQL Infant Scales

	Physical functioning	Physical symptoms	Emotional functioning	Social functioning
*Physical functioning*				
1–12 months (*n* = 50)				
13–24 months (*n* = 95)				
*Physical symptoms*				
1–12 months (*n* = 50)	0.83			
13–24 months (*n* = 95)	0.85			
*Emotional functioning*				
1–12 months (*n* = 50)	0.75	0.61		
13–24 months (*n* = 95)	0.69	0.74		
*Social functioning*				
1–12 months (*n* = 50)	0.59	0.65	0.45	
13–24 months (*n* = 95)	0.63	0.73	0.64	
*Cognitive functioning*				
1–12 months (*n* = 50)	0.60	0.62	0.52	0.89
13–24 months (*n* = 95)	0.30	0.39	0.51	0.64

Pearson’s product–moment correlation coefficients and all *P* values of these coefficients were less than 0.01

### Feasibility

Missing items were independent of each other in the 1–12 months version of PedsQL-I (*P* = 0.35), but there were significant differences in the rate of missing values between items in the 13–24 months version of PedsQL-I (*P* = 0.002) (Additional file 1: Table S1). Some participants did not report the items of the PedsQL-I Cognitive Functioning Scale, for example, “Difficulty pointing to his/her body parts when asked,” “Difficulty naming familiar objects,” and “Difficulty repeating words.” Many participants reported that the items of the PedsQL-I were intelligible.

### Reliability

The scales were internally consistent for participants with infants aged 1–12 months and 13–24 months (Chronbach’s coefficient α 0.88–0.98 and 0.85–0.97, respectively) (Table 2). We distributed retest questionnaires to 66 participants of which 56 (84.8%) were returned. Three retest questionnaires were excluded because participants had mistakenly completed the incorrect version of PedsQL-I in the first test. Fifty-three questionnaires (80.3%) were analyzed for test–retest reliability. In a comparison of respondent characteristics for retest and non-retest participants (Fisher’s exact test or Welch’s t test), retest participants tended to report that their child had acute disease at the first test. There was no significant difference between the initial scale scores of the PedsQL-I and the retest scores (Table 4). The intra-class correlation coefficients between the initial test and the retest all exceeded 0.40, indicating moderate to high agreement.

**Table 4:** Test–retest reliability of the Japanese version of the PedsQL Infant Scales

	N = 52								
	*n*	Mean	SD	95%CI		t^a^	*P* value ^a^	ICC^b^	*P* value^b^
				Upper	Lower				
*Infants aged 1–12 months*									
Physical functioning	23	1.93	18.25	− 5.96	9.82	0.51	0.62	0.71	< 0.01
Physical symptoms	23	0.25	9.97	− 4.06	4.56	0.12	0.90	0.85	< 0.01
Emotional functioning	23	− 1.99	11.12	− 6.80	2.82	− 0.86	0.40	0.73	< 0.01
Social functioning	23	2.72	14.08	− 3.37	8.81	0.93	0.36	0.73	< 0.01
Cognitive functioning	23	− 3.26	11.44	− 8.21	1.69	− 1.37	0.19	0.70	< 0.01
Physical health summary	23	0.73	11.11	− 4.07	5.53	0.32	0.76	0.84	< 0.01
Psychosocial health summary	23	− 3.28	7.65	− 6.59	0.02	− 2.06	0.05	0.80	< 0.01
Total	23	-0.25	8.79	− 4.05	3.55	− 0.14	0.89	0.85	< 0.01
*Infants aged 13–24 months*									
Physical functioning	29	0.38	14.40	− 5.10	5.86	0.14	0.89	0.49	< 0.01
Physical symptoms	28	1.07	16.81	− 5.45	7.59	0.34	0.74	0.65	< 0.01
Emotional functioning	29	− 2.19	9.10	− 5.65	1.27	− 1.30	0.21	0.86	< 0.01
Social functioning	28	0.00	8.50	− 3.30	3.30	0.00	1.00	0.56	< 0.01
Cognitive functioning	27	0.34	6.66	− 2.30	2.97	0.26	0.79	0.77	< 0.01
Physical health summary	28	0.39	13.59	− 4.88	5.67	0.15	0.88	0.66	< 0.01
Psychosocial health summary	29	− 1.28	6.35	− 3.69	1.14	− 1.08	0.29	0.85	< 0.01
Total	29	-0.32	7.94	− 3.34	2.70	− 0.22	0.83	0.81	< 0.01

SD, standard deviation

^a^Paired t test

^b^Intra-class correlation coefficients between the initial test and retest scores in a one-way random effects model

### Validity

With respect to factor validity, the convergent correlation coefficient of the scales varied from 0.26 to 0.77, and the discriminative correlation coefficient of the scales from 0.07 to 0.64. The scaling success rate ranged from 80 to 100% (Table 5). Because a considerable portion of children had the same best score (100) they took up a large weight in the computation of Cronbach’s alpha, test–retest ICC, and correlations. Therefore, we also calculated Cronbach’s alpha, test–retest ICC, and correlations for the subset of participants without the best score of 100 (Additional file 1: Table S2).

**Table 5:** Factorial validity of the Japanese version of the PedsQL Infant Scales

	*N* = 145				
	No. of items per scale	Convergent validity (range of correlations)	Discriminative validity (range of correlations)	Scaling success^a^	Scaling success rate^b^
Physical functioning	9	0.31–0.72	0.14–0.63	36/36	100.0%
Physical symptoms	10	0.34–0.62	0.17–0.59	32/40	80.0%
Emotional functioning	12	0.35–0.73	0.07–0.60	46/48	95.8%
Social functioning	5	0.67–0.74	0.27–0.61	20/20	100.0%
Cognitive functioning	9	0.55–0.77	0.08–0.64	33/36	91.7%
Physical health summary	19	0.26–0.66	0.16–0.56	17/19	89.5%
Psychosocial health summary	26	0.34–0.70	0.10–0.57	24/26	92.3%

^a^Number of convergent correlations significantly higher than discriminant correlations divided by total number of correlations

^b^Scaling success rate is the previous column as a percentage

Validation for known-groups showed that the Physical Health Summary score was sensitive to both acute and chronic disease, the Psychosocial Health Summary score was sensitive to neither acute nor chronic disease, and the total score was sensitive to only acute disease (Table 6). Although the Psychosocial Health Summary score and the total score were insensitive to chronic illness, the results showed a 3.0- and 5.3-point reduction to chronic illness, respectively.

**Table 6:** Known-groups validity of the Japanese version of the PedsQL Infant Scales

	*N* = 145					
	PedsQL Infant Scales					
	Physical health summary		Psychosocial health summary		Total	
	β^a^	*P* value	β^a^	*P* value	β^a^	*P* value
Acute disease	− 7.50	0.01	− 2.93	0.14	− 4.71	0.03
Chronic disease	− 8.70	0.049	− 3.00	0.32	− 5.28	0.12

^a^Non-standardized regression coefficients calculated from linear mixed model controlling child’s age, gender, parent’s age, gender, and K6 score as fixed effects and study institutions (i.e., day care centers and the pediatric clinic) as a random effect. Therefore (for example), PedsQL Infant Scales Total score among infants with acute disease is 4.71 points lower than among those without acute disease

With respect to concurrent validity, correlation coefficients between the PedsQL-I and the PedsQL-T were as follows: Physical Health Summary scores (0.61), Psychosocial Health Summary scores (0.60), and total scores (0.74) (Table 7).

**Table 7:** Concurrent validity of the Japanese version of the PedsQL Infant Scales

		*N* = 43		
		PedsQL Generic Core Scales Toddler Version		
		Physical health summary	Psychosocial health summary	Total
PedsQL Infant Scales	Physical health summary	0.61**	0.48**	0.67**
	Psychosocial health summary	0.51**	0.60**	0.63**
	Total	0.66**	0.54**	0.74**

Pearson’s product–moment correlation coefficients to assess that Physical Health Summary, Psychosocial Health Summary, and the total score on PedsQL Infant Scales were positively correlated with the same scale scores on PedsQL Generic Core Scales

^**^P < 0.01

The PedsQL-I scores were convergent and discriminative valid against the PedsQL-T, with a Pearson’s correlation coefficient for each score as follows: Physical Functioning (0.94), Physical Symptoms (0.64), Emotional Functioning (0.83), and Social Functioning (0.44) (Table 8). Different from our hypothesis, the PedsQL-I Social Functioning Scale correlated better with the PedsQL-T Emotional Functioning Scale than with the PedsQL-T Social Functioning Scale.

**Table 8:** Convergent and discriminative validity of the Japanese version of the PedsQL Infant Scales

		*N* = 43		
		PedsQL Generic Core Scales Toddler Version		
		Physical functioning (Cronbach’s α = 0.72)	Emotional functioning (Cronbach’s α = 0.82)	Social functioning (Cronbach’s α = 0.83)
PedsQL Infant Scales	Physical functioning	0.94***	0.61**	0.32
	Physical symptoms	0.64**	0.63**	0.26
	Emotional functioning	0.73**	0.83***	0.40*
	Social functioning	0.43	0.53**	0.44*

Correlation coefficients divided by Cronbach’s α of each subscale on PedsQL Generic Core Scales Toddler Version calculated in the present study

*P < 0.05. **P < 0.01. ***P < 0.001

Relative validity of the PedsQL-I total score and the Physical Summary score are presented (Table 9). The PedsQL-I was better able to distinguish healthy infants from ill infants than the PedsQL-T with regard to the total score (relative efficiency [RE] = 1.74) and the Physical Summary score (RE = 5.43). Contrary to our expectation, the PedsQL-I Psychosocial Summary score was less able to distinguish healthy infants from ill infants than the PedsQL-T (RE = 0.85).

**Table 9:** Relative validity of the Japanese version of the PedsQL Infant Scales

	*N* = 43		
	Infants aged 19–24 months		
	t value	Toddler version	Ratio of the squared t values
	Infant Scales		
Physical health summary	− 2.12	− 0.91	5.43
Psychosocial health summary	− 1.78	− 1.94	0.85
Total	− 2.12	− 1.61	1.74

Regarding transitional validity of the PedsQL total score, the differences between the estimated score of the PedsQL-I and those of the PedsQL-T at the transition point were 1.0 for the mean (95% confidence interval (CI) − 6.2 to 7.2, *P* = 0.77). The PedsQL Physical Summary score also showed that the mean difference of the estimates at the transition point was − 2.1 and the CI spanned across zero (95% CI − 12.7 to 5.9, *P* = 0.71). Similarly, the two estimates of the PedsQL Psychosocial Summary score had no significant mean difference (3.2, 95% CI − 2.2 to 8.7, *P* = 0.25). Regarding the boundary at age 12.5 months, the Total, Physical Summary and Psychosocial Summary scores also showed no significant mean differences (− 2.6, 95% CI − 10.8 to 5.5, *P* = 0.53; − 0.3, 95% CI − 11.3 to 10.5, *P* = 0.95; and − 4.5, 95% CI − 11.6 to 2.6, *P* = 0.21), respectively.

## Discussion

This study shows that the Japanese version of the PedsQL-I is a feasible, reliable, and satisfactory measure of HRQOL for infants aged 1–24 months.

High ceiling effects were observed; however, they had no substantial effect on psychometric properties, as can be judged from the computations done on the subset of infants without the best score. Especially-high ceiling effects observed with the Social Functioning Scale and the Cognitive Functioning Scale were likely due to participants evaluating their own children, who were healthy enough to live at home or in day care. Parents tend to acknowledge their children’s receptiveness to their own cues positively. Relatively high ceiling effects for the Social and Cognitive Functioning Scales have been reported previously for infants and children [11, 25, 26]; in the present study, however, they were particularly high. The Social, Cognitive and Emotional Functioning Scales construct the Psychosocial Health Summary in the PedsQL-I. The Psychosocial Health Summary had no high ceiling effect. It is possible that infants’ psychosocial aspects are broad and undifferentiated.

Some participants did not report items 6, 7, and 8 in the Cognitive Functioning Scale in the 13–24 months version. For item 6; “Difficulty pointing to his/her body parts when asked,” it might have been difficult to articulate an answer, which then resulted in not being able to answer the subsequent items 7; “Difficulty naming familiar objects” and item 8; “Difficulty repeating words.” This may be partly because very unhealthy (e.g. hospitalized) infants did not participate in this study, and partly because of individual variability in development, i.e., some parents might consider these items less applicable to their own children. However, the overall missing rate was only 0.7%, suggesting good feasibility. Rather, we cannot omit these items. The larger number of items in the PedsQL-I than the PedsQL-T would be necessary to understand infant HRQOL with broad and undifferentiated aspects.

The internal consistency reliability of the total score and all five subscale scores of the PedsQL-I was good and the test–retest reliability was also satisfactory. Together, this suggested sufficient reliability of the PedsQL-I. Proof of the validity of our results was assessed by convergent validity, discriminative validity and scaling success rate. Scaling success rate for all subscales exceeded 80%, which ensured factorial validity [27]. Validation for known-groups showed that the Physical Health Summary score was sensitive to both acute and chronic disease, however contrary to our hypothesis, the Psychosocial Health Summary score was sensitive to neither acute nor chronic disease. This study did not include infants who were hospitalized or institutionalized. Therefore, it is possible that the difference between the groups would be smaller than expected. This may have caused the lack of statistical difference. Another reason might be the lack of power due to the small sample size as a result of an unexpectedly low questionnaire response rate.

Regarding concurrent validity, each score of the PedsQL-I showed a high correlation to PedsQL-T. However, the PedsQL-I Social Functioning Scale correlated better with the PedsQL-T Emotional Functioning Scale than with the PedsQL-T Social Functioning Scale. For children in infancy and early childhood, emotional functioning is consistently based on the parent–child relationship. On the other hand, social relationships for children expand rapidly. Reflecting this, in contrast to the PedsQL-I Social Functioning scale, which asks about parents' perceptions of their infants' reactions to their parents, the PedsQL-T Social Functioning scale asks about parents' perceptions of their children's interactions with other children. We considered this to be a reason for the result.

Relative validity reported a ratio of squared t values of 1.74, which justifies the use of this scale in infants up to 24 months of age. Further, there was no difference in the score of intercept between age transition. To our knowledge, the present study is the first to show that it is appropriate to use the total score of the PedsQL-I as a continuous value between the age groups 1 and 2 years. In longitudinal HRQOL studies that follow patients from infancy, the PedsQL-I should be useful to study HRQOL at first, and the results could be readily taken over by the PedsQL-T as an infant matures.

A strength of the present study was that we included participants from diverse socio-demographic backgrounds. However, our sample was limited to participants from Tokyo and information on non-participants was not available. Thus, the results maybe not be generalizable to all of Japan. Second, the small sample size is a limitation of this study. In particular, it was difficult to recruit a defined number of known-group infants for each age group due to the unpredictably large number of infants with an acute or chronic disease and an unexpectedly low questionnaire response rate.

Third, the high ceiling effects observed in the present study might limit the usefulness of the scale in discerning health improvements in healthy or mildly ill infants. Fourth, we could not use comparable supporting measures (i.e. TAPQOL) to match each domain of PedsQL-I because Japanese versions have not been developed. Fifth, acute and chronic diseases of the infants in the present study were parent-reported and not verified. Further research will evaluate the use of the PedsQL-I in well-defined infant populations including hospitalized and institutionalized infants.

The present study taught us several points about infant HRQOL research. One notable point is that 50.0–71.1% of children in different age groups had an acute disease. Children in these age groups tend to develop acute conditions easily but recover within a few days. Therefore, it might be more reasonable to use a 7-day recall rather than a 1-month recall. Further, we assumed the factor structure of the PedsQL-I original version. As our sample size did not allow us to conduct exploratory or confirmatory factor analysis, we used multi-trait analysis, but this may suffer from single-rater bias. Parents have been thought of as the best reporters to assess their children’s HRQOL [28]. In the future, however, additional forms of QOL evaluation, such as dyad comparison of mother- and father-report, and multi-view, which includes the view of surrounding family members and child health professionals, as well as the growing infants’ own perspective, might bring further understanding to this challenging field.

## Conclusions

The PedsQL-I is suitable for assessing health-related quality of life in healthy infants aged 1–24 months. We expect that the PedsQL-I should be useful to study HRQOL in a variety of infants (with various diseases) and for conducting follow-up studies conjoint with the PedsQL-T. Future research will help expand our understanding of and develop strategies to improve infant HRQOL.

## Supplementary Information

**Additional file 1.** Percentages of Missing Items and Subgroup Analyses in a Subset without the Best Score of 100.

## Data Availability

The datasets generated and/or analyzed during the current study are available from the corresponding author on reasonable request.

## Abbreviations

HRQOL: Health-related quality of life
ICC: Intraclass correlation coefficient
ITQOL: Infant and Toddler Quality of Life Questionnaire
JWV: James W. Varni
K6: Kessler-6
PedsQL: Pediatric quality of life inventory
PedsQL-I: Pediatric Quality of Life Inventory Infant Scales
PedsQL-T: Pediatric Quality of Life Inventory Generic Core Scales Toddler Version
RE: Relative efficiency
TAPQOL: TNO/AZL Preschool Children Quality of Life Questionnaire
PRO: Patient-reported outcomes
