# Supplementary material for: Reliability and validity of the Japanese version of the Pediatric Quality of Life Inventory Infant Scales
Source: J Patient Rep Outcomes. 2022 Jan 29;6:10. doi: 10.1186/s41687-022-00416-3 (PMC8800985; doi:10.1186/s41687-022-00416-3)
Supplement: Supplementary file 1 — Additional file 1. Percentages of Missing Items and Subgroup Analyses in a Subset without the Best Score of 100. [file 41687_2022_416_MOESM1_ESM.docx]

Supplementary Table S1. Percentages of missing items in each scale

|  | 1-12 months | (*N*=50) | 13-24 months | (*N*=95) |
| --- | --- | --- | --- | --- |
|  | Number of  missing items | (%) | Number of  missing items | (%) |
| Physical Functioning |  |  |  |  |
| 1 | 1 | 2.0 | 2 | 2.1 |
| 2 | 1 | 2.0 | 2 | 2.1 |
| 3 | 1 | 2.0 | 4 | 4.2 |
| 4 | 1 | 2.0 | 2 | 2.1 |
| 5 | 1 | 2.0 | 1 | 1.1 |
| 6 | 1 | 2.0 | 2 | 2.1 |
| 7 | - | - | 2 | 2.1 |
| 8 | - | - | 2 | 2.1 |
| 9 | - | - | 4 | 4.2 |
| Physical Symptoms |  |  |  |  |
| 1 | 1 | 2.0 | 2 | 2.1 |
| 2 | 1 | 2.0 | 3 | 3.2 |
| 3 | 1 | 2.0 | 3 | 3.2 |
| 4 | 1 | 2.0 | 3 | 3.2 |
| 5 | 1 | 2.0 | 2 | 2.1 |
| 6 | 0 | 0.0 | 3 | 3.2 |
| 7 | 1 | 2.0 | 2 | 2.1 |
| 8 | 2 | 4.0 | 2 | 2.1 |
| 9 | 1 | 2.0 | 3 | 3.2 |
| 10 | 1 | 2.0 | 3 | 3.2 |
| Emotional Functioning |  |  |  |  |
| 1 | 1 | 2.0 | 4 | 4.2 |
| 2 | 1 | 2.0 | 3 | 3.2 |
| 3 | 0 | 0.0 | 2 | 2.1 |
| 4 | 0 | 0.0 | 3 | 3.2 |
| 5 | 0 | 0.0 | 2 | 2.1 |
| 6 | 2 | 4.0 | 5 | 5.3 |
| 7 | 1 | 2.0 | 4 | 4.2 |
| 8 | 1 | 2.0 | 3 | 3.2 |
| 9 | 1 | 2.0 | 2 | 2.1 |
| 10 | 0 | 0.0 | 3 | 3.2 |
| 11 | 1 | 2.0 | 2 | 2.1 |
| 12 | 0 | 0.0 | 3 | 3.2 |
| Social Functioning |  |  |  |  |
| 1 | 0 | 0.0 | 3 | 3.2 |
| 2 | 0 | 0.0 | 4 | 4.2 |
| 3 | 0 | 0.0 | 4 | 4.2 |
| 4 | 0 | 0.0 | 4 | 4.2 |
| 5 | - | - | 3 | 3.2 |
| Cognitive Functioning |  |  |  |  |
| 1 | 2 | 4.0 | 4 | 4.2 |
| 2 | 2 | 4.0 | 4 | 4.2 |
| 3 | 2 | 4.0 | 4 | 4.2 |
| 4 | 1 | 2.0 | 5 | 5.3 |
| 5 | 2 | 4.0 | 5 | 5.3 |
| 6 | - | - | 7 | 7.4 |
| 7 | - | - | 7 | 7.4 |
| 8 | - | - | 7 | 7.4 |
| 9 | - | - | 5 | 5.3 |

Supplementary Table S2. Cronbach’s alpha, test-retest ICC, and correlations between subscales in a subset of infants without the best score of 100.

|  |  |  |  |  |  | *N*=145 |
| --- | --- | --- | --- | --- | --- | --- |
|  | Cronbach’s α | ICC | Correlations^a^ |  |  |  |
|  |  |  | Physical Functioning | Physical Symptoms | Emotional Functioning | Social Functioning |
| Infants aged 1–12 months |  |  |  |  |  |  |
| Physical Functioning | 0.90 | 0.65 |  |  |  |  |
| Physical Symptoms | 0.87 | 0.84 | 0.82 |  |  |  |
| Emotional Functioning | 0.93 | 0.72 | 0.75 | 0.61 |  |  |
| Social Functioning | 0.97 | 0.61 | 0.58 | 0.64 | 0.45 |  |
| Cognitive Functioning | 0.74 | 0.59 | 0.64 | 0.66 | 0.54 | 0.94 |
| Physical Health Summary | 0.93 | 0.84 |  |  |  |  |
| Psychosocial Health Summary | 0.94 | 0.80 |  |  |  |  |
| Total | 0.95 | 0.85 |  |  |  |  |
|  |  |  |  |  |  |  |
| Infants aged 13–24 months |  |  |  |  |  |  |
| Physical Functioning | 0.88 | 0.32 |  |  |  |  |
| Physical Symptoms | 0.90 | 0.61 | 0.82 |  |  |  |
| Emotional Functioning | 0.89 | 0.85 | 0.65 | 0.71 |  |  |
| Social Functioning | 0.82 | 0.34 | 0.60 | 0.71 | 0.62 |  |
| Cognitive Functioning | 0.66 | 0.71 | 0.31 | 0.41 | 0.54 | 0.70 |
| Physical Health Summary | 0.95 | 0.63 |  |  |  |  |
| Psychosocial Health Summary | 0.93 | 0.84 |  |  |  |  |
| Total | 0.97 | 0.81 |  |  |  |  |

ICC: intraclass correlation coefficient

^a^Pearson’s product–moment correlation coefficients and all *P*-values of these coefficients were less than 0.05.
